# Strategies and limitations in app usage and human mobility

**DOI:** 10.1038/s41598-019-47493-x

**Published:** 2019-07-29

**Authors:** Marco De Nadai, Angelo Cardoso, Antonio Lima, Bruno Lepri, Nuria Oliver

**Affiliations:** 1 Vodafone Research, Paddington Central, London, W2 6BY UK; 2 Mobs Lab, Fondazione Bruno Kessler, Via Sommarive 18, 38123 Povo, TN Italy; 3 Department of Information Engineering and Computer Science, University of Trento, Via Sommarive, 9I, 38123 Povo, TN Italy

**Keywords:** Computational science, Information technology, Scientific data, Software, Statistics

## Abstract

Cognition has been found to constrain several aspects of human behaviour, such as the number of friends and the number of favourite places a person keeps stable over time. This limitation has been empirically defined in the physical and social spaces. But do people exhibit similar constraints in the digital space? We address this question through the analysis of pseudonymised mobility and mobile application (app) usage data of 400,000 individuals in a European country for six months. Despite the enormous heterogeneity of apps usage, we find that individuals exhibit a conserved capacity that limits the number of applications they regularly use. Moreover, we find that this capacity steadily decreases with age, as does the capacity in the physical space but with more complex dynamics. Even though people might have the same capacity, applications get added and removed over time. In this respect, we identify two profiles of individuals: app *keepers* and *explorers*, which differ in their stable (keepers) vs exploratory (explorers) behaviour regarding their use of mobile applications. Finally, we show that the capacity of applications predicts mobility capacity and vice-versa. By contrast, the behaviour of *keepers* and *explorers* may considerably vary across the two domains. Our empirical findings provide an intriguing picture linking human behaviour in the physical and digital worlds which bridges research studies from Computer Science, Social Physics and Computational Social Sciences.

## Introduction

Recent studies on mobility and social interactions suggest that cognitive constraints, rather than time, might be the primary cause of the limited number of places and friends that people maintain at any point in their life time^1–5^. Thanks to the wide adoption of smartphones and the proliferation of mobile applications (apps), almost any human need –from entertainment to social connection or productivity– can be satisfied by at least one of the two million mobile apps available in the major app stores^6^. As a consequence, people spend an increasing amount of time on their smartphones, reaching an average of 3 hours per day in 2018^7^ and triggering debates about their effect on human cognition and attention^8–10^. Interestingly, despite this ever-growing *digitisation* of human life and availability of apps, people tend to exploit a small set of repeatedly used apps^11^. Is it the case then, that human behaviour on digital devices exhibits similar dynamics and constraints as those found in the *physical* world?

Similarly to mobility, we know that human behaviour on mobile phones has regular daily rhythms^12^ that coexist with a *bursty* and highly heterogeneous usage^11^, where most of the applications struggle to stay relevant longer than a fortnight^13^. The existing literature has leveraged these findings to predict short-term dynamics (e.g., next used app), understand the relationship with user’s actions and context, or recommend apps^14–19^. Only a few studies have tried to characterise the statistical properties of the adoption and use of mobile applications^11,20^, relying however on fixed observation windows, which hinder the temporal variations of used and abandoned apps over time. Such a limitation has been mainly caused by the absence of data describing long-term human behaviour on mobile phones. The available data is indeed usually based on a few weeks of network traffic generated by both foreground and background applications, which sometimes are automatically launched by the phone without the user’s will^21^.

In this paper, we analyse the use of foreground applications to compare human behaviour between the digital and physical worlds. To the best of our knowledge, this is the first research effort to study app usage alongside with mobility in a large population over six months. In a modern society with high adoption of smartphones, understanding applications usage has both theoretical and practical implications in a variety of fields from the design of digital services to human behaviour understanding and modelling.

## Results

We study six months of pseudonymized data collected through an Android application installed in hundreds of thousands of devices in a European country. The application allows users to get individual usage reports, as well as contribute to the overall improvement of the services provided to them, such as diagnose and improve network connectivity and quality. Upon installation, the app –which runs in the background– asks its users for explicit consent to record at regular intervals the state of the device, its usage and the context where it is used (e.g., GPS coordinates). We consider only data generated by users having GPS locations covering at least 80% of the hours of each individual, and having application usage data for the entire period. We characterise human mobility through an individual’s set of locations, where *locations* are defined as places where people stop for at least 15 minutes. To uniformly analyse human behaviour on mobile devices, we consider only those apps available in the Google Play Store, which is the main store for Android devices. After the filtering, the data consists of 415,000 users that stop in 138 million locations and use 69,000 different applications that were launched for a total of around 1 billion times. We refer to the Methods and Supplementary Information (SI) for further details about the processing and sampling approaches.

As mentioned before, previous literature has mainly investigated application usage from either limited and controlled contexts, or short-term passive collection of network traffic, which limit the ability to capture app usage. Network-level measurements, in particular, include data generated by both background and foreground applications, which makes it very hard to analyse actual human behaviour^21^. Thus, we here begin by describing some statistical properties of the foreground application usage.

**Figure 1:**
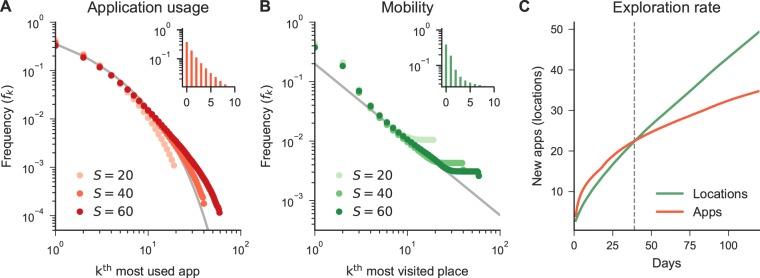
Aggregated statistics of app usage and mobility. (**A**) Truncated power-law plot showing the frequency *f*_*k*_ of the most used *k* applications. We show in red three users with a different number of distinct applications *S*. The grey line shows that the empirical data is well approximated by ${f}_{k} \sim {(k+{k}_{0})}^{-\alpha }exp(\,-\,k/c)$, where *α* = 1.19 ± 0.01, *k*_0_ = 1.14 ± 0.07, and *c* = 8.32 ± 0.75. The inset illustrates that more than 40% of the time people are found using the first two apps. (**B**) Zipf’s law plot showing the frequency *f*_*k*_ of the most visited *k* locations of users with different number of distinct total locations *S*. The grey line shows that the empirical data is well approximated by ${f}_{k} \sim {(k)}^{-\alpha }$, where *α* = 1.27 ± 0.01. The inset illustrates that more than 40% of the time people are found in the first two locations. (**C**) Average number of new apps and locations discovered by users over time.

During the entire six-month period people used on average a total of 27 different apps, mostly belonging to the *Communication* and *Social* categories (see Supplementary Information (SI) Fig. S2(A)). The *Communication* category includes all the applications that allow users to send messages to other people (e.g., WhatsApp, Messenger), while the *Social* category includes Social Network Apps (e.g., Pinterest, Facebook, Instagram). The usage frequency and time spent on apps by an individual is heavily skewed. We find that the app usage is well described by a truncated power-law, where the frequency *f* of the *k*^*th*^ most visited location is well approximated by: ${f}_{k} \sim {(k+{k}_{0})}^{-\alpha }exp(\,-\,k/c)$, with exponent *α* = 1.19 ± 0.01, *k*_0_ = 1.14 ± 0.07 and a cut off value *c* = 8.32 ± 0.75. Thus, the time spent by people on phones is mostly focused on few apps, although users possess at least 26 applications (see SI Fig. S7). Figure 1(A) shows the distribution of application usage for people with different number of distinct apps *S*. Similar results are obtained for background applications, where the distribution is even more skewed towards the first app (see SI Fig. S5(E)). Mobility is well described by a power-law distribution ${f}_{k} \sim {k}^{-\alpha }$ with *α* = 1.27 ± 0.01, which is compatible to the results found in literature^22^ (*α* = 1.2 ± 0.1). While the distributions between mobility and application usage are different, the exponent *α* of the power laws show a similar tendency towards the skewed use of time in locations and apps.

Nevertheless, human behaviour evolves over time. Figure 1(C) shows the number of new locations and apps that people discover over time. We model the total number of apps as $L{(t)}_{apps}\propto {L}_{0}{t}^{{\gamma }_{1}}$ where *t* is the time, *L*_0_ is a normalisation constant and *γ*_1_ a growing coefficient, and the number of locations as $L{(t)}_{mob}\propto {L}_{0}{t}^{{\gamma }_{2}}$. We find *γ*_2_ = 0.64 and *γ*_1_ = 0.41, revealing a surprising fact: people explore new locations over time at a much faster pace than they add new apps (in particular, after 39 days from Fig. 1(C)). Thus, while people tend to use a small set of applications, they also continuously explore new applications over time (at a slower rate than new locations, though).

To explain this apparent contradiction, we characterise the mobility and app usage through the *activity space* and *the app space*. In mobility, the activity space^4^ is defined as the set $Mob{S}_{i}(t)=[{{\mathfrak{l}}}_{1},{{\mathfrak{l}}}_{2},\,\ldots ,\,{{\mathfrak{l}}}_{n}]$ of stop locations an individual *i* visits at least twice and spends on average more than 10 min per week over a time-window *t*. In a similar fashion, we define the *app space* as the set of applications $App{S}_{i}(t)=[{{\mathfrak{a}}}_{1},{{\mathfrak{a}}}_{2},\,\ldots ,\,{{\mathfrak{a}}}_{n}]$ that are used at least twice by the user *i* in a time window *t*. The app space describes the set of apps that are used at any point in time by an individual. As both application usage and mobility are *bursty*^12,23^, too short and too long time windows might hide dynamics and erroneously identify spurious behaviour. Thus, similarly to previous works^4^ we use a time window *d* = 20 weeks long. Note that we tested the sensitivity of the window size and found no significant differences.

### Capacity and activity of app usage

**Figure 2:**
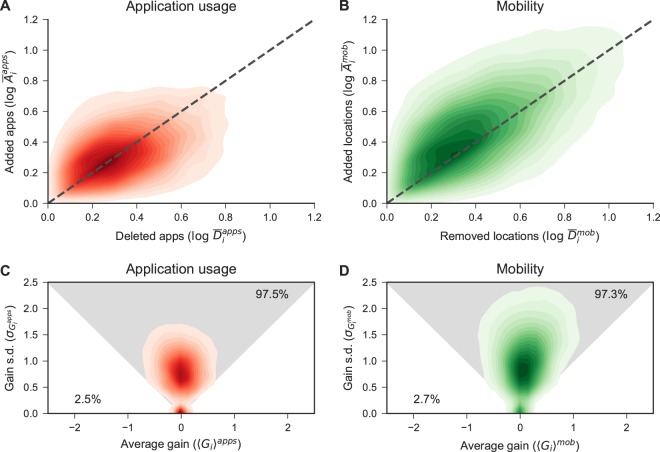
Capacity and activity of application usage and mobility for all the individuals. (**A,B**) Density plots $\rho (\mathrm{log}\,{\bar{D}}_{i},\,\mathrm{log}\,{\bar{A}}_{i})$ for users with $\mathrm{log}\,{\bar{A}}_{i} > 0$ and $\mathrm{log}\,{\bar{D}}_{i} > 0$ in both application usage and mobility. (**C,D**) Average gain of apps (locations) versus the standard deviation of the app (locations) gain. The grey area corresponds to individuals with a conserved size of applications (locations): ${\overline{G}}_{i}\le {\sigma }_{{G}_{i}}$ for user *i*. 97.5% of the users has a conserved capacity in application usage, and 97.3% of them has a conserved capacity in mobility.

The activity and app spaces allow to observe the evolution of the set of preferred locations and applications over time. First, for each user *i* we define the *app capacity* as the number of distinct apps used by the user in a time window *t*: ${C}_{i}^{apps}(t)=|{{\rm{AppS}}}_{{\rm{i}}}(t)|$ to observe how the number of familiar apps changes over time. Then, we model the relative average capacity across the sample population as: ${\bar{C}}^{apps}(t)/\langle \bar{C}{\rangle }^{apps}={\alpha }^{apps}+{\beta }^{apps}t$. We find that the app capacity is constant in time, as the slope of this linear relation does not markedly differ from zero (*β*^*apps*^ = 0.0023). We also test the alternative hypothesis where the capacity would be a consequence of the high heterogeneity of the sample population: with some people shrinking it and others expanding it over time. For each individual *i* we measure the *app gain* ${G}_{i}^{apps}(t)={A}_{i}^{apps}(t)-{D}_{i}^{apps}(t)$, defined as the difference between the number of added ${A}_{i}^{apps}(t)$ and removed ${D}_{i}^{apps}(t)$ apps over two time windows (e.g. [*t*, *t* + *d*) and [*t* + *r*, *t* + *r* + *d*) with a slide *r*), and we also define the *net gain* as the average absolute gain over time divided by the standard deviation of it $|{\langle {G}_{i}\rangle }^{apps}|/{\sigma }_{{G}_{i}^{apps}}$. People having net gain within one standard deviation (s.d.) are expected to be consistent with 〈*G*_*i*_〉 = 0, while people with $|{\langle {G}_{i}\rangle }^{apps}|/{\sigma }_{{G}_{i}^{apps}} > 1$ increase or decrease their net gain over time. We find that 97.3% of the people in our data have $|{\langle {G}_{i}\rangle }^{apps}|/{\sigma }_{{G}_{i}^{apps}}\le 1$, thus exhibiting a conserved *app capacity* (see Fig. 2(C)).

We also computed the same metrics for mobility. We find that the mobility capacity *C*^*mob*^ is constant over time (*β*^*mob*^ = 0.0064) and that 97.5% of the users have a net gain that does not significantly differ from zero $(|{\langle {G}_{i}\rangle }^{mob}|/{\sigma }_{{G}_{i}^{mob}}\le 1)$ (see Fig. 2(D)) These results are in agreement with the literature^4^.

Interestingly, this empirical study uncovers a remarkable similarity between mobility and the application usage domains. Figure 2(A) depicts the relationship between the average number of added and removed apps, while Fig. 2(B) shows the same relationship for the mobility domain.

By observing the apps that are kept and dropped from the app space the most, we can shed light on the shift in the interests of our users. For each user and app, we computed the average percentage of weeks an app is kept in the app space once a user adopts it. We refer to this measure as the *persistence* of the apps. The categories kept for a longer time are *Social* and *Communication*, which supports the view of smartphones being used mainly for social connection. Differently, the Art & Design, Events and Games categories have the lowest persistence, highlighting the strong relation of some apps with temporal local events and trends. On average, an app persists for 40% of the observation window. A subset of apps (around 10%) is kept almost continuously while more than 17% of them are kept for just one time window and dropped afterwards (see SI Fig. S9). In particular, two apps are on average continuously used and kept in the app space: WhatsApp and Facebook, probably due to the network effect, i.e. as the number of people using a service increases so does the value of using it^24,25^. These apps have been reported to have amongst the highest levels of adoption in smartphone users in the country of study^26^. Conversely, proprietary and niche apps such as Samsung Keyboard and Secure Folder have low persistence and hence are dropped from the app space very frequently. The persistence of apps in the app space is strictly related to their use, while in mobility this relation is less clear for locations that are rarely visited (see SI Fig. S10). We refer the reader to SI Table S4 and Fig. S9 for further details about the persistence analysis of apps and locations.

Our results indicate that the app capacity is conserved for most individuals. However, it might be a direct consequence of time constraints, as common sense would suggest. People have limited time to allocate to different activities on a daily basis. Thus, we break application usage in daily modules and shuffle it through two types of randomisation. For example, given the temporal sequence of app usage for two different users, the local randomisation shuffles the temporal order of the sequence of apps for one user, while the global randomisation shuffles the sequences across all the users. We refer to the Methods Section for further details. We find that capacity is constant even after shuffling the individual time series with both types of randomisation. Moreover, the two-sided Kolmogorov-Smirnov (KS)^27^ test rejects the hypothesis that the two random time series have a similar underlying distribution to the original one (KS-local: 0.55 *p*-value < 0.001, KS-global: 0.98 *p*-value < 0.001). As the KS distance is lower in the case of local randomisation: these results suggest that the app capacity is not just a consequence of time constraints but an inherent property of human behaviour.

Interestingly, we find that human behaviour in the app space is very similar to that of the mobility space, as shown in Fig. 2. We find a significant and positive correlation (Pearson’s r 0.16, *p*-value < 0.001) between mobility and app capacity, but also between the individual number of new locations and new apps (Pearson’s r 0.11, *p*-value < 0.001) (see SI Fig. S1). These positive and significant correlations might be a consequence of a trade-off between mobile phone usage and mobility, where people tend to decrease their mobility when they use the phone for longer times and vice-versa. Hence, we break individual behaviour into one-day modules, where each module describes the cumulative time spent in locations and the cumulative time spent on apps in the day. Then, we compare human dynamics through the total time spent in visited locations and the total time spent on applications in three different time windows (*i.e*., daily, weekly, monthly). We do not find any strong negative correlation between these variables, which would imply the existence of a trade-off between mobile phone usage and human mobility. On the contrary, by comparing the number of visited locations with the number of app launches we do find a slightly positive correlation. In other words, the higher the mobility, the higher the usage of apps is (see SI Section S2).

### Keepers and explorers

Previous work has found that people can be grouped in two groups through the regularity of their behaviour: people who tend to behave according to constant and repetitive habits and those who tend to change their behaviour over time^1,28,29^. This result has been found, under different names, in previous work regarding social connections^1^ and mobility^29^. However, to the best of our knowledge, the literature has not yet explored this dichotomy in the behaviour regarding the use of applications.

**Figure 3:**
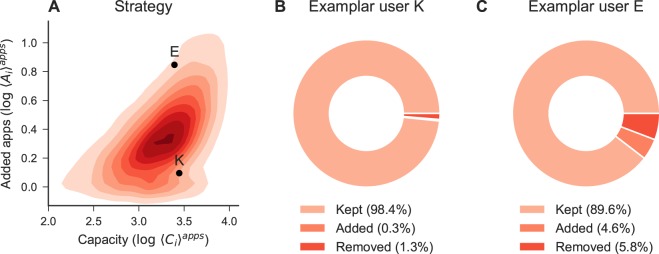
Variability of strategy. (**A**) Density plot showing between the app capacity and the new apps discovered. We randomly select two users with similar capacity, but different strategy, which we name E and K. (**B**) Individual K – an app keeper – has an average of less than 1% added and removed apps. (**C**) Individual E – an app explorer – explores more than user K.

Capacity influences the rate of new apps and locations discovered over time. While the growth exponent of apps is *γ*_1_ = 0.41 for the entire population, an average user with average capacity 〈*C*_*i*_〉 = 80 discovers new apps at a much larger rate (*γ*_1_ = 0.53). Similarly, individuals with 〈*C*_*i*_〉 = 20 discover new locations at a much slower pace than people with 〈*C*_*i*_〉 = 80 (*γ*_2_ = 0.58 vs *γ*_2_ = 0.78). However, we note that users with the same app capacity might have a very different rate of new apps discovered. To illustrate this point, we randomly select two users, namely *K* and *E*, from the set of people who have similar app capacity but exhibit a very different number of newly discovered apps. Figure 3 shows that user *K* used roughly the same applications during the entire period of study, whereas user *E* added new apps in the app space and removed some of them as well, thus maintaining constant capacity. Similarly to previous work^1^, we encode this *strategy* through the ratio between the number of newly adopted apps and the user’s average capacity ${R}_{i}^{apps}={\langle {A}_{i}\rangle }^{apps}/{\langle {C}_{i}\rangle }^{apps}$. We define application *explorers* to be those users with ${R}_{i}\gg \beta$ and application *keepers* to be those users with ${R}_{i}\ll \beta$, where *β* corresponds to the average behaviour over all the users. We compute the same measure in the physical space using the mobility capacity and the new locations added to the users’ *activity space*, defining ${R}_{i}^{mob}={\langle {A}_{i}\rangle }^{mob}/{\langle {C}_{i}\rangle }^{mob}$. Previous work has defined the explorers-keepers dichotomy in mobility through the radius of gyration, which is the radius of the circumference that encloses most of the locations usually visited by an individual. Thus, such a definition is about the size of the geographic space explored by people. However, our definition of explorers vs keepers is about the rate of adoption of new locations that are visited regularly by individuals. Therefore, our definition is consistent with our concept of explorers vs keepers in the applications domain and also to previous work in the case of social connections^1^.

By defining explorers from the distribution of *R*^*apps*^ as those with *R*^*apps*^ higher than the 80^th^ percentile and returners as those with *R*^*apps*^ lower than the 20^th^ percentile, we observe that explorers adopt on average one app every 28 weeks (${\bar{A}}^{apps}=0.72$), while keepers adopt a new app at a much slower rate (${\bar{A}}^{apps}=0.04$). The *strategy* in app usage is also reflected in the persistence of mobile apps in the app space. We indeed find that apps are more persistent among keepers, who tend to use the apps they adopt for 77% of the observation windows, on average. On the contrary, explorers are less loyal to new apps and keep their apps for only 60% of the observation window (see SI Fig. S10). The two distributions are well separated and do not come from the same underlying distribution (KS: 0.49 *p*-value < 0.001).

When we apply the same concept to mobility, the results are surprising. As common sense would suggest, discovering and visiting new locations costs more energy than discovering and installing new mobile applications, even if some of them are not free. However, the number of adopted and discarded locations is larger than the number of apps. On average, mobility explorers embrace a new familiar location every 17 weeks (${\bar{A}}^{mob}=1.16$), while keepers adopt a new location at a much slower rate (${\bar{A}}^{mob}=0.11$). Similarly, keepers retain a new location for 61% of their time while new locations are less persistent for explorers who maintain them for 40% of the observation window (see SI Fig. S10). The two distributions are well separated and do not come from the same underlying distribution (KS: 0.52 *p*-value < 0.001). Once again, this result shows that people’s behaviour in the digital world is more repetitive than in the physical one. Although social relations and tightly coupled with mobility^3,30^, most of the social relations today might be managed by only a few apps such as Facebook, Whatsapp and Messanger, which are the most kept applications in the app space (see SI Table S2).

As both capacity and activity are correlated across domains, we also compare strategies between application usage and mobility. First, we classify individuals in one of the three classes in the app domain: namely *explorer*, *keeper* and *other*. This last class describes all the “average” behaviour within two standard deviations of *R*^*apps*^. We do the same according to their mobility strategy. Then, we use a Random Forest Classifier with 20 estimators where the independent variable is each user’s app strategy *R*^*apps*^, and we predict the corresponding class in the mobility domain. We fit the model in a Stratified 5-fold Cross-Validation fashion to avoid over-fitting. While the model shows severe imbalance over the average class (*other*), we find that it is possible to predict the mobility strategy using as input the application strategy with a micro-averaged F1-score of 0.53, with a baseline of 0.44. We obtain similar results when we train using the labels of the users’ mobility strategy *R*^*mob*^ to predict their app strategy (micro-averaged F1-score: 0.54, with a baseline of 0.44). Even though the strategies correlate across the app and mobility domains, we find that it is very challenging to predict one from the other.

### Age-dependency of app usage and mobility

In this section, we shift our focus to demographic differences in the users’ app and mobility behaviour. Our data contains age information for 92.6% of the users who range from 18 to 68 years old with *μ* = 39 years and *σ* = 12 years, as shown in the SI Fig. S6.

**Figure 4:**
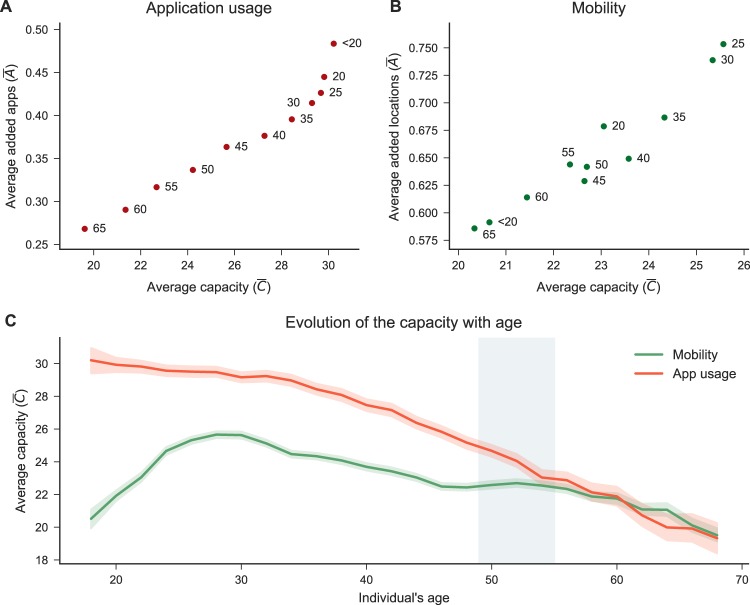
Age-variations of capacity and novelty. (**A**,**B**) Average capacity and novelty for groups of users of different age. (**A**) suggests a strong dependence of applications with age, while (**B**) is less clear, especially for people less than 35 years old. (**C**) shows that the average app capacity steadily decreases when age increases, while mobility capacity increases until around 28 years, then decrease until 46 where it remains constant, to decrease again from 56 years onward.

We analyse the relationship between the age of the users in our data set, and their app and mobility capacities. As perhaps expected, we find a strong negative correlation between age and app usage. Figure 4(C) shows that younger people –those aged between 18 and 24 years– have the highest average number of applications in their app space. From 20 years of age onward, the average app capacity declines monotonically. Interestingly, mobility behaves very differently depending on the age of our users. As illustrated in Fig. 4(C), in early adulthood individuals seem to increase their mobility capacity from around 20 to 26 preferred locations. Then, a slow decrease starts until approximately the age of 48, where the capacity plateaus for a few years to then decrease again monotonically with age. This result might be related to life events that have an impact on people’s mobility. A recent survey^31^ involving 300,000 Britons, shows that the point in life where people are the most dissatisfied is 49, while the peak of satisfaction is around 30 years. Surprisingly, the results of this survey correspond to the beginning of the plateau in average mobility capacity and the highest point of mobility capacity in our data, respectively.

Additionally, we found that people’s strategy is influenced by their age. In the app space, the older the people are, the less prone they are to discover and add new apps in the app space (Pearson-r −0.99, *p*-value <0.001). In the case of mobility, such a relationship is weaker but still very significant (Pearson-r −0.57, *p*-value < 0.001). Similarly, the persistence of apps in the app space is highly related to people’s age (Pearson-r: 0.65, *p*-value: < 0.001) while in mobility the correlation is not significant. Figure 4(A) shows an evident tendency of older people to have a smaller capacity and exploration rate than younger people, while Fig. 4(B) shows that this relationship is less clear, especially for individuals younger than 35 years old. We analyse the relationship between age and the persistence of an app in the app space and a location in the activity space. We find that age is significantly correlated with app persistence (Pearson’s r 0.64, *p*-value < 0.001) while mobility is not (Pearson’s r −0.31, *p*-value > 0.001). We model for each age bin *b* the number of discovered apps over time as $L{(t)}_{apps}\propto {L}_{0}{b}_{1}{t}^{{\gamma }_{1}}$, and the number of discovered locations over time as $L{(t)}_{mob}\propto {L}_{0}{b}_{2}{t}^{{\gamma }_{2}}$, where *L*_0_ is a normalisation constant, *γ*_1_ and *γ*_2_ are growing coefficients, and *t* is the time. Similarly to what we have reported in the previous sections, we find from the standardised coefficients that the exploration of new apps is slower than locations (*γ*_1_ = 0.385, *γ*_2_ = 0.589). However, we also discover that age is negatively correlated with the discovery rate in the app domain (*b*_1_ = −0.113) while this correlation is weaker for mobility (*b*_2_ = 0.009) (see SI Fig. S8C,D). Next, we analyse the distribution of strategies in our users grouped by age –in bins of five-years. We find that in mobility the ratio of explorers and returners is almost constant for all groups (see SI Fig. S3(B)). However, in the case of apps we observe that young people have a low percentage of returners and a high rate of explorers (~12% and 28% respectively), and the percentage of returners increases with age (the last age-group has ~27% of returners and ~19% explorers) (see SI Fig. S3(A)), which is an intuitive finding.

## Discussion

In this paper, we have studied whether the inherent properties of human behaviour found in social relations and mobility also apply to mobile application usage. We have compared the statistical properties of app usage and mobility through the analysis of a large data set containing the mobile app behaviour of hundreds of thousands of individuals over six months. We have found that, despite the high heterogeneity of application usage, individuals can be described through their *app capacity* and their *app space*. The former expresses the conserved and limited number of apps an individual uses in any point of their life –which remarkably is almost the same value as their mobility capacity. The latter represents the number of novel apps adopted over time. We have found that app capacity is not a direct consequence of time constraints but an individual behaviour that might be connected with our cognitive limits, in line with the Dunbar’s social brain hypothesis^32^, which fixes to around 150 the number of friends people can maintain at any point in their life. In this respect, it is interesting to note that recent preliminary results on online content consumption are aligned with our results^5^.

However, people are not all alike. Previous work has found two main strategies grouping people into those who tend to exploit the same items over time, and those who tend to explore new items, where items are relationships, places or actions. While researchers have referred to these strategies with different terms –*e.g*. returners and explorers^29^ and social keepers and explorers^1^– the findings are consistent across different domains. Here, we have found empirical evidence for the first time that people also exhibit this exploration vs exploitation dichotomy in their app usage behaviour. Moreover, we also provide a novel definition of mobility keepers and explorers, which is consistent in the app and social domains^1^. Surprisingly, the strategies do not always match across domains: keepers in the app domain could be explorers in the physical space and *vice-versa*. Additionally, contrarily to common sense, we also find that the number of adopted and discarded locations is larger than the number of added and discarded apps: mobility explorers adopt on average more than one new location every 17 weeks, whereas app explorers adopt on average one new app every 28 weeks.

Capacity and strategy are correlated with age in both the applications and mobility domains. Although we did not analyse the data for the same individuals over multiple decades, our result suggests that capacity is a stable property over short-term periods of time but would evolve (and mainly decrease) with age. Age-specific life events and goals profoundly influence our behaviour, especially in the social domain^33^. Also, cognitive abilities –which typically decline with age– play a role in shaping our interests, actions and our decisions regarding how we spend our time^34^. Although we identify a significant positive correlation between the mobility and the app capacity and also between new locations and new apps, these correlations, are not due to a trade-off between mobile phone usage and mobility. Thus, we do not find clear evidence of the impact of mobile phone usage on mobility.

A quantitative understanding of human behaviour on digital devices is of uttermost importance to interpret the profound and fast changes happening in our contemporary society. Together, our results not only extend to the mobile app domain previous empirical results on social relations^1,35^, purchase behaviour^36–38^, online communities^39^ and human mobility^3,4^, but also shed new light on the interplay between the physical and the digital worlds.

## Methods

We analyse data concerning application usage and GPS coordinates. We use data from 400,000 users in a European country for six months, ending in July of 2018. From the raw data we obtain two different data streams of the same users to support our analysis: the *User Locations* data stream, composed of (user ID, date, time, latitude, longitude); and the *User Application Usage* data stream, composed of (user ID, date, application name, aggregated time spent, number of times opened). For a subset of the users with also have some limited *User Demographic* data, consisting of their ID and self-reported age for 92.6% of the users. All users are adults (18+ years old).

The entire study design and conduct were carried out and approved through Vodafone’s Data Analysis code of conduct, signed by all authors. To safeguard personal privacy, all data is pseudonymised and collected with full informed consent, in agreement with existing data privacy and data protection regulations and analysed according to Vodafone code of conduct. All methods were carried out in accordance with relevant guidelines and regulations.

All user IDs are hashed and randomised to preserve anonymity. All results and insights showed in this manuscript are aggregated over thousands of individuals.

### User locations

This data has been obtained from the GPS coordinates that are collected from either actual GPS measurements with an error of less than 30 m, or through a WiFi look-up performed by the device’s operating system. We do so to avoid spurious detection of locations. We filtered out all users whose locations are available less than 80% of the time, to ensure that we have enough data to characterise their mobility appropriately. The resulting data has 383,422 users with mobility information, with a median number of GPS coordinates per day per user of 96. We extract the *stop events* with an algorithm based on Hariharan and Toyama^40^, where a stop event is defined as a temporal sequence of GPS coordinates in a radius of Δ*s* meters where the user stayed for at least Δ*t* minutes. The algorithm, its optimisation and its complexity are explained in details in the SI. The presented results are for Δ*s* = 50 meters and Δ*t* = 15 minutes, parameters similar to the literature^4,29^. For each user, we define *stop locations* as the sequences of *stop events* that can be considered part of the same place. To determine a *stop location* from *stop events* we use the DB-scan (*minPoints*, *ε*) algorithm^41^ that groups points within *ε* = Δ*s*−5 meters of distance to form a cluster with at least *minPoints* = 1 stop event (see SI Section S1 for more details). In sum, we characterise the users’ mobility by their sequence of *stop locations*.

### Application usage

This data contains the timestamp, number of launches and the screen time of all the applications that are launched by the users. This allows the analysis of the real behaviour of the users, without the well-known problems of network traffic data^21^. To further highlight the differences between background and foreground applications we refer to the SI Fig. S5. We focus our analysis on applications that are downloadable from the Google Play store, thus excluding vendor-specific applications. This allows investigating people’s behaviour uniformly across devices. At this moment, the store has 11 main categories, namely *Business*, *Communication*, *Fitness*, *Game*, *Lifestyle*, *Music*, *Personalization*, *Photography*, *Reading*, *Social*, *Tools*, and *Travel*. We filter out apps belonging to the *Personalization* and *Tools* to avoid most of the manufacturer-specific lock screen apps and custom launchers (e.g. com.htc.launcher).

To reduce the noise of the data, we also exclude those app launches that lasted less than one second, which might be related to apps opened by mistake. This rule of thumb choice exclude just a small portion of data without affecting the overall results (see the SI).

The resulting data has 92,943 users with app usage information, with a median number of 7 different apps launched per day per user.

### Global and local randomisation

We test whether the app capacity *C*^*apps*^ is a consequence of time constraints by applying two randomisation techniques previously proposed for the mobility space^4^. Let *X* and *Y* be two users with daily usage of apps *D*_*X*_ = [*d*_*X*,*t*_, *d*_*X*,*t*+1_, …, *d*_*X*,*t*+*n*_] and *D*_*Y*_ = [*d*_*Y*,*t*_, *d*_*Y*,*t*+1_, …, *d*_*Y*,*t*+*n*_] the two randomisation strategies are:

- *Local*. We permute the order of the sliding observation windows at random. For example we shuffle the two original time-series of *X* and *Y*: *D*_*X*_ = [*d*_*X*,*t*+5_, *d*_*X*,*t*_, …, *d*_*X*,*t*+1_] and *D*_*Y*_ = [*d*_*Y*,*t*_, *d*_*Y*,*t*+*n*_, …, *d*_*Y*,*t*+1_].
- *Global*. We permute an individual’s data across the entire data. For example: *D*_*X*_ = [*d*_*Y*,*t*_, *d*_*X*,*t*+1_, …, *d*_*Y*,*t*+*n*_] and *D*_*Y*_ = [*d*_*Y*,*t*_, *d*_*Y*,*t*+1_, …, *d*_*X*,*t*+*n*_] (Note the shuffle appendix of the last element in the sequence).

### Cross-domain strategy prediction

We predict people’s strategy from the app domain to the mobility domain and *vice-versa* with a Random Forest classifier. The training and evaluation are done in a 5-fold Cross-validation fashion. The classifier has 50 estimators, uses all features with sample bootstrapping and trees are grown until there is one sample per leaf (implemented with the scikit-learn framework^42^ v0.19 using parameters: n_estimators = 50, bootstrap = True, min_samples_leaf = 1, max_features_leaf = None and all other parameters set to default). We also tested for other hyper-parameters with a Grid search strategy without finding significant improvements. We rely on the F1-score as evaluation metric, since it balances the precision and recall of the model. As the task is a multi-class classification with three classes (*explorer*, *keeper* and *other*) the F1-score reported in the manuscript is micro averaged. Results with the macro-averaged F1-score can be found in the SI Table S6. In the same table, we report the results for an alternative model based on a multinomial Logistic Regression and a baseline, which randomly predicts the class by respecting the training set’s class distribution. The Logistic regression model is trained with a lbfgs solver and weights for class imbalance. As shown in the Table, Random Forests deliver the best performance.

## Supplementary information

Strategies and limitations in app usage and human mobility - Supplementary Information

## Data Availability

The studied data is not publicly available. To protect the privacy of the users the data cannot be freely shared. However, they are available to researchers through an agreement with Vodafone Research to respect the code of conduct and criteria of confidentiality and privacy protection. The source code of the stop location algorithm and for some of the plots is available at: https://github.com/denadai2/apps-mobility-capacity-strategy. The source code to repeat the experiments is available on request.
