# Supplementary material for: Strategies and limitations in app usage and human mobility
Source: Sci Rep. 2019 Jul 29;9:10935. doi: 10.1038/s41598-019-47493-x (PMC6662905; doi:10.1038/s41598-019-47493-x)
Supplement: Supplementary file 1 — Strategies and limitations in app usage and human mobility - Supplementary Information [file 41598_2019_47493_MOESM1_ESM.pdf]

Strategies and limitations in app usage and human mobility - Supplementary Information

Marco De Nadai, Angelo Cardoso, Antonio Lima,

Bruno Lepri and Nuria Oliver

1 The stop location algorithm

As described in the main manuscript, the *Users Location* data stream is composed of (user ID, date, time, latitude, longitude).

1.1 Stop events

From a sequence of ordered time events $T = [t_0, t_1, \ldots, t_n] \mid t_j \leq t_i$, a corresponding set of GPS locations $R = [r_0, r_1, \ldots, r_n]$, and a geographical distance function $d(i, j)$, we define a *stop event* as a maximal set of locations $S = [r_i, r_{i+1}, \ldots, r_j] \mid d(r_i, r_j) < \Delta s \wedge t_j - t_i \geq \Delta t \; \forall r_i, r_j \in S$. Then the set of *stop events* is $\mathcal{S} = \{S_i \mid S_i \text{ is a stop event} \wedge r_i \in S_i \wedge r_j \in S_j \wedge i < j\}$. To form a *stop event* we heuristically choose to group locations in a time-ordered fashion. In other words, we aim at finding all those places at most Δs meters large were people stopped for at least Δt minutes. Each *stop event* is composed by at least two locations and the locations can belong only to at most one *stop event*.

To extract *stop events* we base our method on Hariharan and Toyama's work [3]. The algorithm is depicted in Algorithm 1 and can be summarised as follows: for each user, we first order his/her GPS locations by time, followed by selecting groups of GPS sequences with the desired properties to form *stop events*. The `Diameter` function computes the greatest distance between points, while `Medoid` selects the GPS location with the minimum distance to all other points in the set.

The complexity of the *stop event* algorithm [3] is $\mathcal{O}(n^3)$, because of the repeated `Diameter` function that computes a distance matrix, whose complexity is $\mathcal{O}(n^2)$. Thus, we make two optimisations to this basic algorithm in order to improve its complexity:

- Each time we compute `Diameter`(R, *left*, j) we cache the computed distance matrix so that we can use it again whenever we need to compute `Diameter`(R, *left*, $j+1$). This reduces the complexity to $\mathcal{O}(n^2)$.

Algorithm 1: Algorithm for extracting the stop *events* from GPS sequences.

Input: Time-ordered list of a user's raw GPS positions $R = [r_0, r_1, \ldots, r_n]$, their time $T = [t_0, t_1, \ldots, t_n]$, a spatial threshold Δs and a temporal threshold Δt.
Output: The set S of a user's stop events.

```
left = 0; S ← ∅ ;
while left < n do
    right ← minimum j such that t_j ≥ t_left + Δt;
    if Diameter(R, left, j) > Δs;
     then
        left ← left +1;
    end
    else
        right ← maximum j such that j ≤ n and Diameter(R, left, j) < Δs;
        S ← S∪ (Medoid(R, left, right), t_left, t_right) ;
        left ← right +1;
    end
end
```

- We reduce the number of points that are most likely not part of a *stop event*. Thus, we filter out $\forall r_i \mid d(r_{i-1}, r_i) < 10\text{m} \wedge \mid d(r_i, r_{i+1}) < 10\text{m}$, but also those $\forall r_j \mid d(r_{j-1}, r_j) > \Delta s \wedge \mid d(r_j, r_{j+1}) > \Delta s$. Although simple, this heuristics keep the complexity on average around $\mathcal{O}(n)$ and in the worst case $\mathcal{O}(n^2)$.

The `Diameter` algorithm can be further optimised by converting all coordinates to a Cartesian plane, then finding the smallest convex region containing all the points and finally computing the diameter in linear time between the points of the convex hull. However, in this work we choose to have higher accuracy using the original coordinates and defining $d(i, j)$ as the Haversine great-circle distance between i and j. Given the average radius of the Earth r and two points with latitude and longitude φ_1, φ_2 and λ_1, λ_2 respectively, the Haversine distance d between them is:

$$d = 2r \arcsin\left(\sqrt{\sin^2\left(\frac{\varphi_2 - \varphi_1}{2}\right) + \cos(\varphi_1)\cos(\varphi_2)\sin^2\left(\frac{\lambda_2 - \lambda_1}{2}\right)}\right)$$

The Haversine distance does not require to project points to a plane, and it is more accurate both in short and long distances.

1.1.1 Stop locations

For each user, we define *stop locations* as the sequences of *stop events* that can be considered part of the same place. For example: if user A goes many times at the Colosseum in Rome, she could have many *stop events* (*e.g.*, northern entrance, southern

entrance) that can be grouped in a unique *stop location* (*i.e.* the Colosseum). To determine a *stop location* from *stop events* we use the DB-scan [2] algorithm that groups points within $\epsilon = \Delta s - 5$ meters of distance to form a cluster with at least $minPoints = 1$ *event*. The complexity of DB-scan is $\mathcal{O}(n)$. We horizontally scale the computation through different cloud machines thanks to Apache Spark.

Taking as a reference previous work [1, 7, 3] we choose $\Delta s = 50$ meters and $\Delta t = 15$ minutes. We qualitatively noticed that with $\Delta s = 30$ (same as the error threshold for our data filtering) the stop locations are more noisy. Similarly, $\Delta t = 10$ minutes may form some spurious stop locations.

We select $\epsilon = \Delta s - 5$ meters to avoid the creation of an extremely –and incorrect– long chain of sequential *stop events*. Thus, $\epsilon = 45$ meters. However, *stop events* and *stop locations* may be very sensible to the Δs and Δt parameters. Therefore, we repeated our experiments both with $\Delta s = 60$ and $\Delta t = 10$ and we found no significant differences. For this reason, in the next Sections we align our discussion to the existing literature and use $\Delta s = 50$ meters and $\Delta t = 15$ minutes.

2 From applications to mobility

We investigated the relationship between mobile app usage behaviour and mobility by correlating the capacity, activity, and strategy between app usage and mobility. However, temporally aggregated behaviour might hide choices people make at a smaller time scale. Thus, we break down people's behaviour on a daily, weekly and monthly basis and test for any trade-off between the time spent in stop locations and the time spent on different types of apps. For each user i we compute the time spent in visited locations $L_i = [l_{i,1}, l_{i,2}, \ldots, l_{i,n}]$, and the time spent on apps $W_i = [w_{i,1}, w_{i,2}, \ldots, w_{i,n}]$ at the chosen level of temporal aggregation. Then, we concatenate all m users' behaviours: $\Phi = [L_0, L_1, \ldots, L_m]$ and $\Gamma = [W_0, W_1, \ldots, W_m]$ and test through the Kendall's τ [5] three different variables:

- **Raw**: *if an individual spends more time using apps, does (s)he stops for longer time in places?* Defined as: $\tau(\Phi, \Gamma)$.
- **Average behavior**: *if an individual spends more time than what other people on average use apps, does his/her mobility decrease?* Defined as: $\tau(\Phi', \Gamma')$ with $\Phi' = [L'_0, \ldots, L'_m]$, $\Gamma' = [W'_0, \ldots, W'_m]$, $L'_i = L_i - \overline{L_i}$ and $W'_i = W_i - \overline{W}$.
- **Individual behavior**: *if an individual spends exceptionally more time than his/her average or baseline on mobile apps, does his/her mobility suffer?* Defined as: $\tau(\Phi'', \Gamma'')$ with $\Phi'' = [L''_0, \ldots, L''_m]$, $\Gamma'' = [W''_0, \ldots, W''_m]$, $L''_i = \frac{L_i - \overline{L_i}}{\omega_{L_i}}$ and $W''_i = \frac{W_i - \overline{W_i}}{\omega_{W_i}}$.

For a set of pairs (i, j) at time t, the Kendall rank coefficient measures how much the rank of the pair changed from t to $t + 1$. The coefficient is 1 when the ranks are identical, while it is -1 when they are dissimilar. In other words, we expect the Kendall's τ to be

positive and high when application usage is very similar to mobility, while we expect it to be negative in the presence of a trade-off between the two domains. Similarly, we also test this trade-off in the frequency domain, comparing the number of apps launched and the number of visited locations.

Thus, we compare the app usage and mobility dynamics and look for any trade-off or positive correlation between these two domains. A strong negative correlation between the two domains echoes previous studies linking smart-phone addiction to negative outcomes such as obesity [6], while a strong positive correlation mean people use phones especially when they move, or with a scale-free dynamic.

Table S1 summarises the results of such an analysis. As depicted in the Table, we do not find any strong negative correlation between these variables, which would represent the existence of a trade-off between mobile phone usage and human mobility. On the contrary, in the frequency domain we do find a slight positive correlation in the Average and Raw behaviours. In other words, when people launches apps more than what other people do, they increase also the mobility.

In summary, in this manuscript we find that capacity is positively correlated between the two domains, but users might adopt different strategies in each domain. Empirical results have shown that intense use of the phone does not necessarily predict well-being [4]. Similarly, our results on the trade-off suggest that people, on average, do not decrease (increase) their physical mobility (as measured by the time spent in visited places) because of the high (low) phone usage. We only found a slight positive correlation on the Average and Raw behaviours, which might be a consequence of the intense phone usage during commuting [8]. However, the lack of agreement between the Raw, Average and Individual's average might suggest that these two domains reflect different aspects of human behaviour. We leave the investigation of this hypothesis to future work.

Table S1: Kendall's τ correlation between daily, weekly, and monthly number of locations and time spent on applications. The Raw, Average, and Individual's average behaviour are sightly but significantly positive correlated. (**) stands for p-value < 0.001.

**Granularity**	**Raw**	**Average**	**Individual's average**
**Time domain**			
Daily	-0.001^{**}	-0.084^{**}	0.007^{**}
Weekly	0.015^{**}	-0.022^{**}	0.045^{**}
Monthly	0.006^{**}	-0.019^{**}	0.070^{**}
**Frequency domain**			
Daily	0.066^{**}	0.115^{**}	0.026^{**}
Weekly	0.136^{**}	0.153^{**}	0.064^{**}
Monthly	0.158^{**}	0.157^{**}	0.084^{**}

3 Additional figures

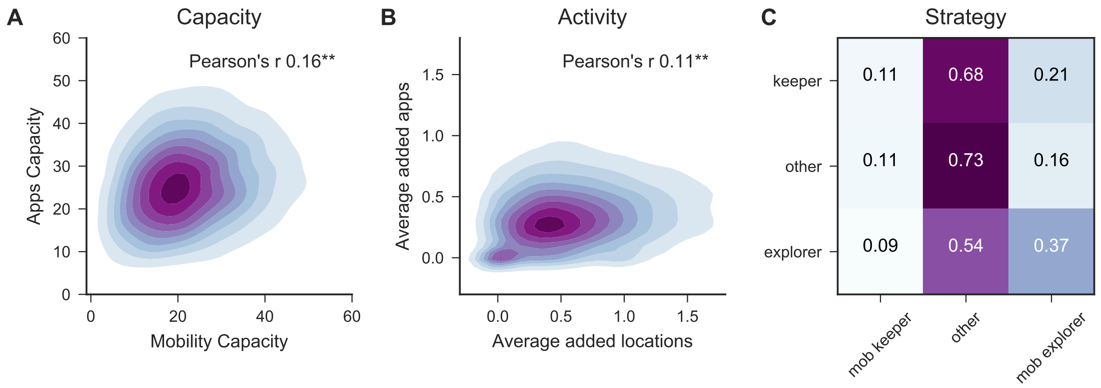

Figure S1: Match between the mobility and digital domain. (A) Density plot showing a positive an significant correlation between users' mobility capacity and application capacity. (B) Density plot between users' mobility and application activity, showing a significant and positive correlation, although grouped in two dense regions. (C) Math between an individual's label in the digital domain and the label assigned in the mobility domain. This confusion matrix shows that labels does not often match across domains. (**) stands for p-value < 0.001.

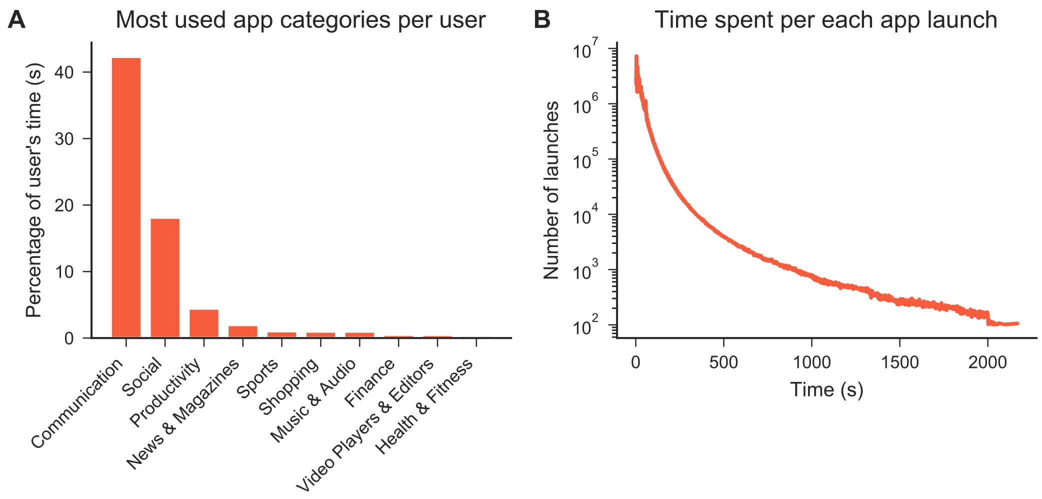

Figure S2: Description of applications launch in our data. (A) The total time spent for each applications' category is very focused on a few categories. (B) PDF Distribution of the time spent on apps per each apps launch.

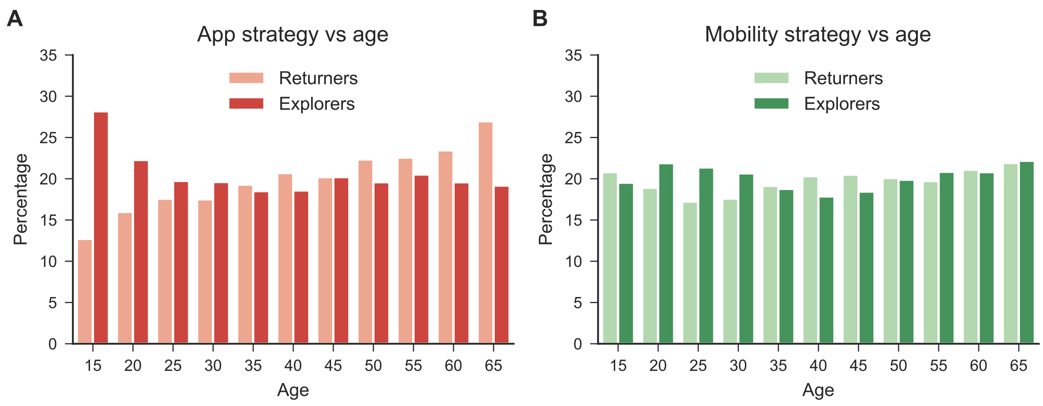

Figure S3: Strategy of people. (A) Percentage of mobility explorers and keepers per each age bin of 5 years. (B) Percentage of app explorers and keepers per each age bin of 5 years.

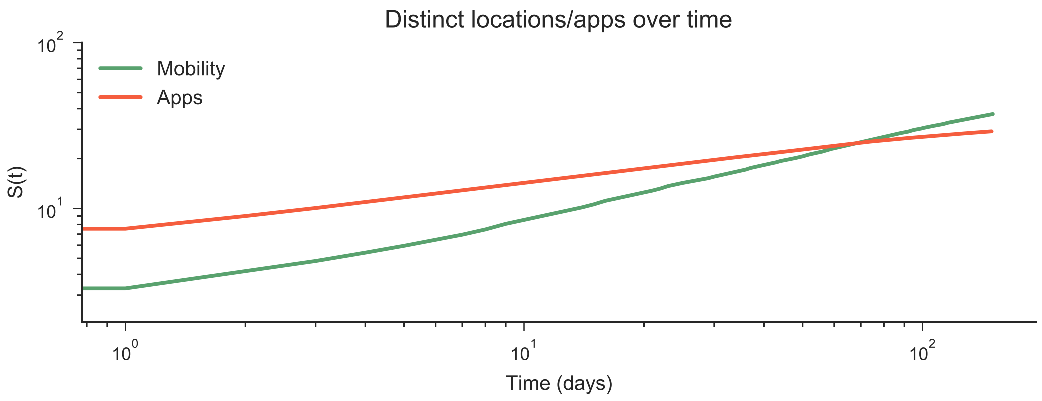

Figure S4: The cumulative number of locations and apps per user over time.

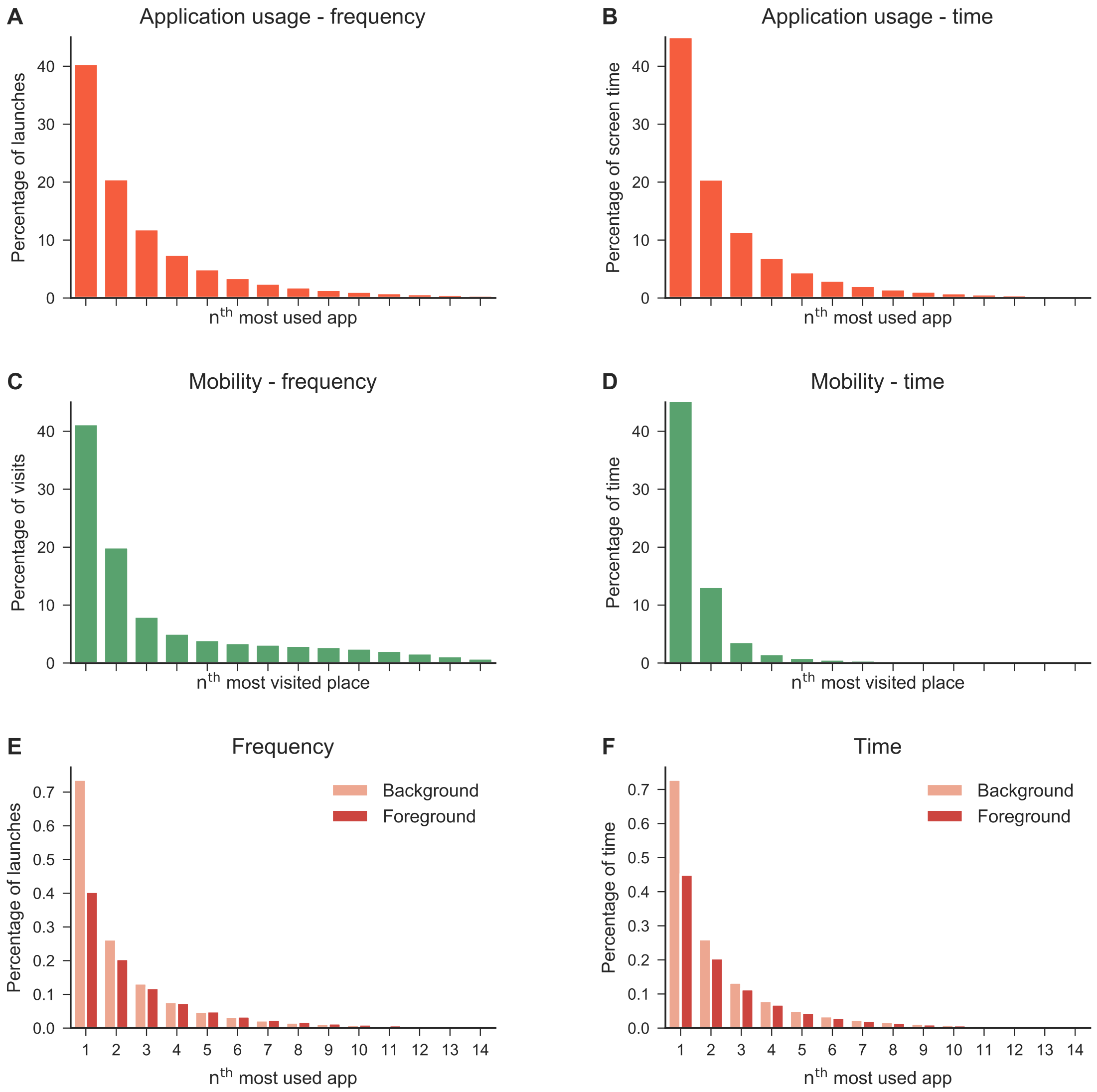

Figure S5: Descriptive statistics in both frequency and time domains, for mobility and application usage. (A) The application usage in terms of frequency. The most used app occupies almost 40% of users' time. (B) The application usage in terms of frequency. The most used app occupies more than 40% of users' time. (C) The frequency of visits for the first 15 locations. The most visited place occupies almost 40% of users' time. (D) The time spent on places for the first 15 locations. (E-F) We compare the frequency of background and foreground apps. The background applications are more skewed than foreground ones in both time and frequency domain.

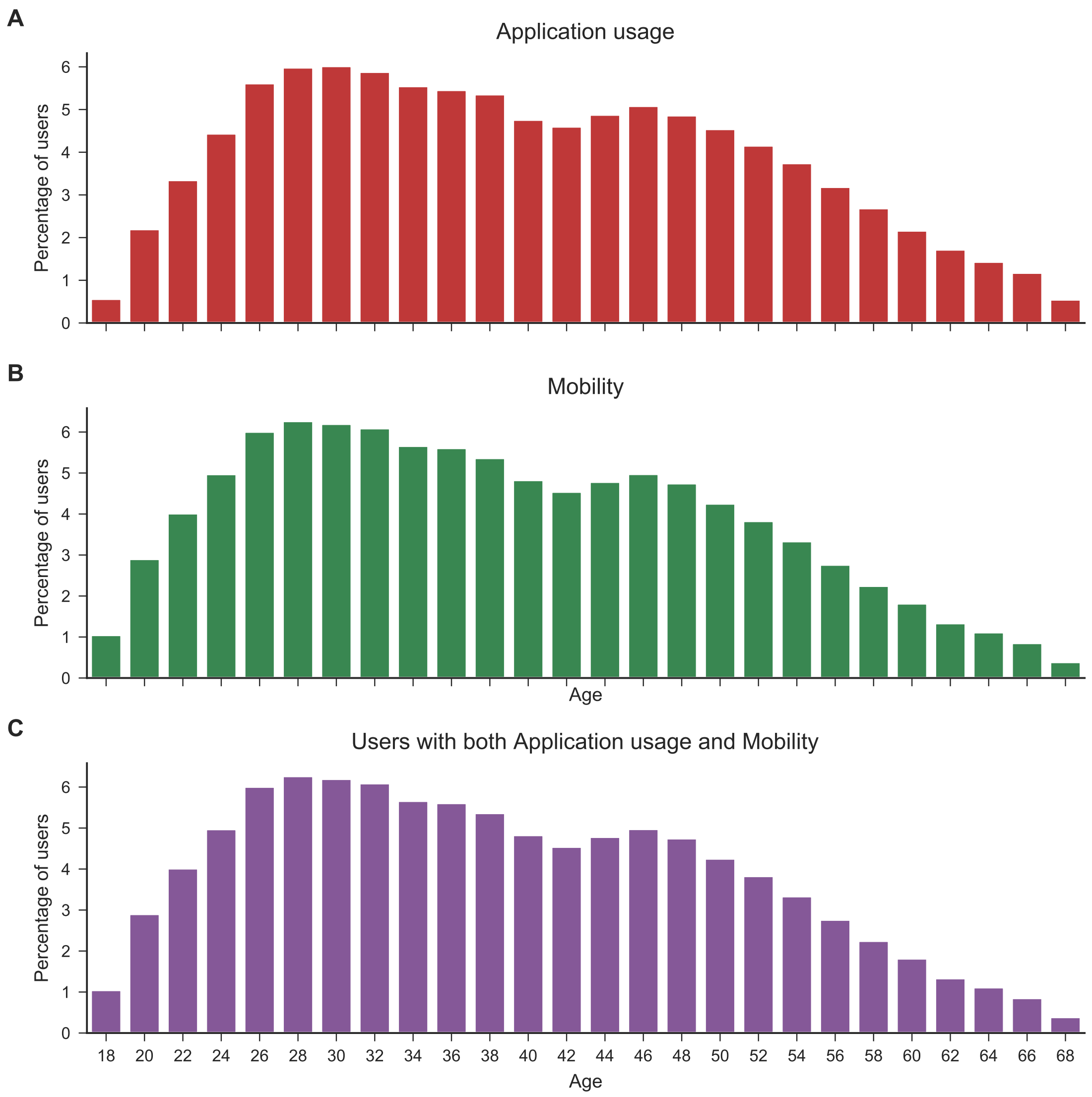

Figure S6: Age distribution in our dataset (in bins of two years). (A) Distribution of ages for those users where we have mobility information. (B) Distribution of ages for those users where we have application usage information. (c) Distribution of ages for those users where we have both mobility and application usage information.

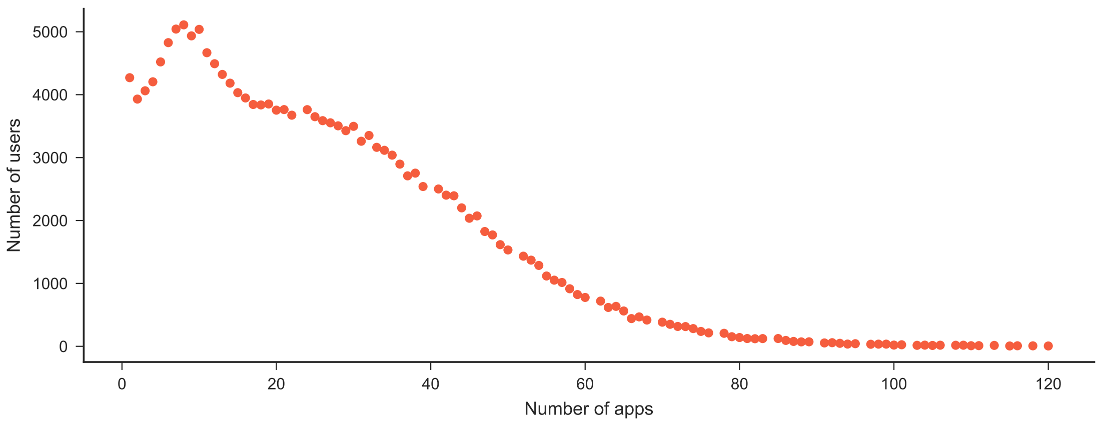

Figure S7: Distribution of the distinct number of apps installed by the users in our data. Note that this number is computed through the applications used at least once in our time period.

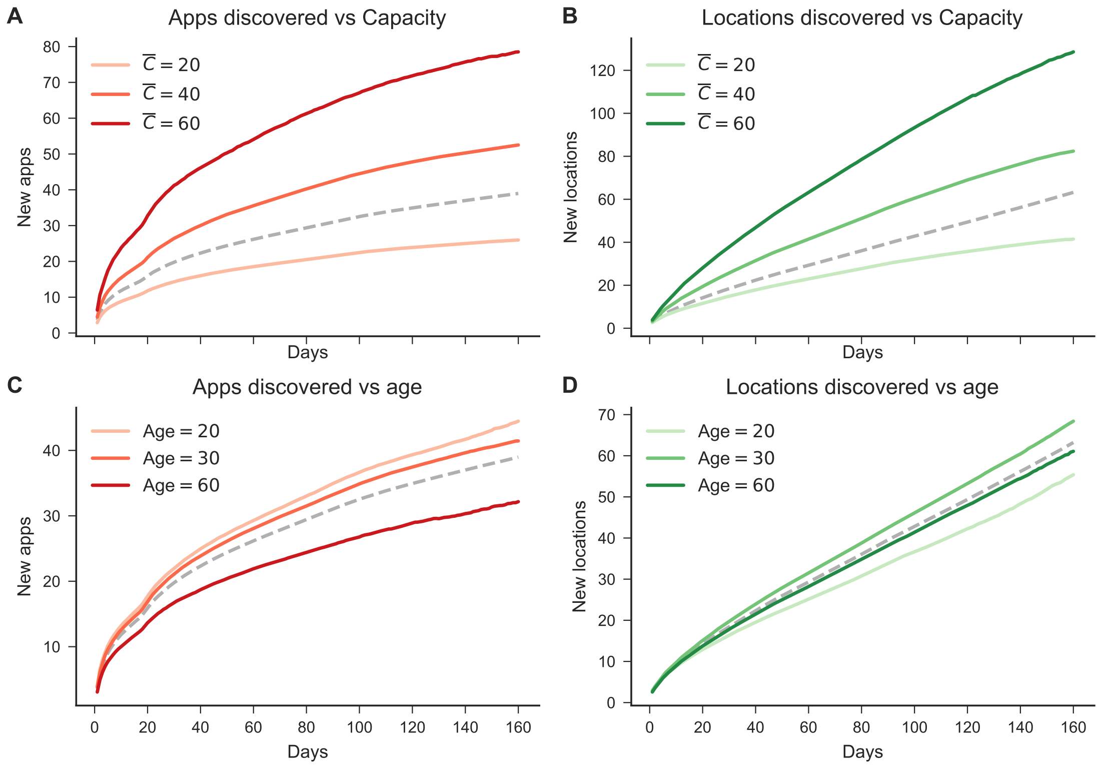

Figure S8: Average number of new apps and locations discovered by users over time. The grey dashed line shows the average across the population. (A-B) Apps (A) and locations (B) discovered over time for users with different average capacity $\overline{C}$; (C-D) apps (C) and locations (D) discovered over time for users with different ages.

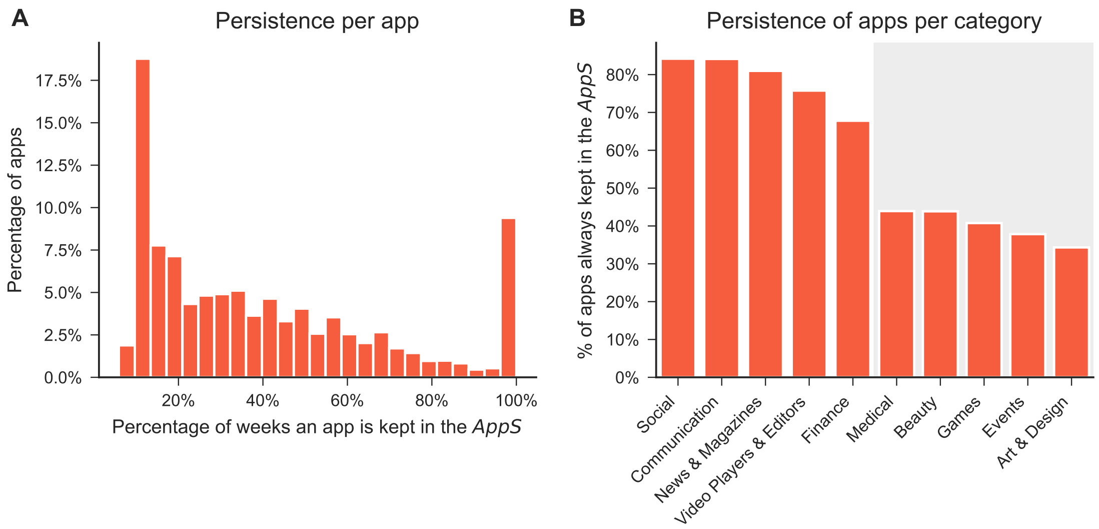

Figure S9: Percentage of time an app is kept in the app space once it enters. (A) Distribution of the percentage of time an app is kept in the app space. We note that around 10% of the apps are continuously used and kept in the app space. (B) The apps that are kept for longer mostly belong to the Social and Communication categories, which are kept for more than 80% of the weeks. On the contrary, apps in the Games, Events and Art & Design categories are kept for very few weeks.

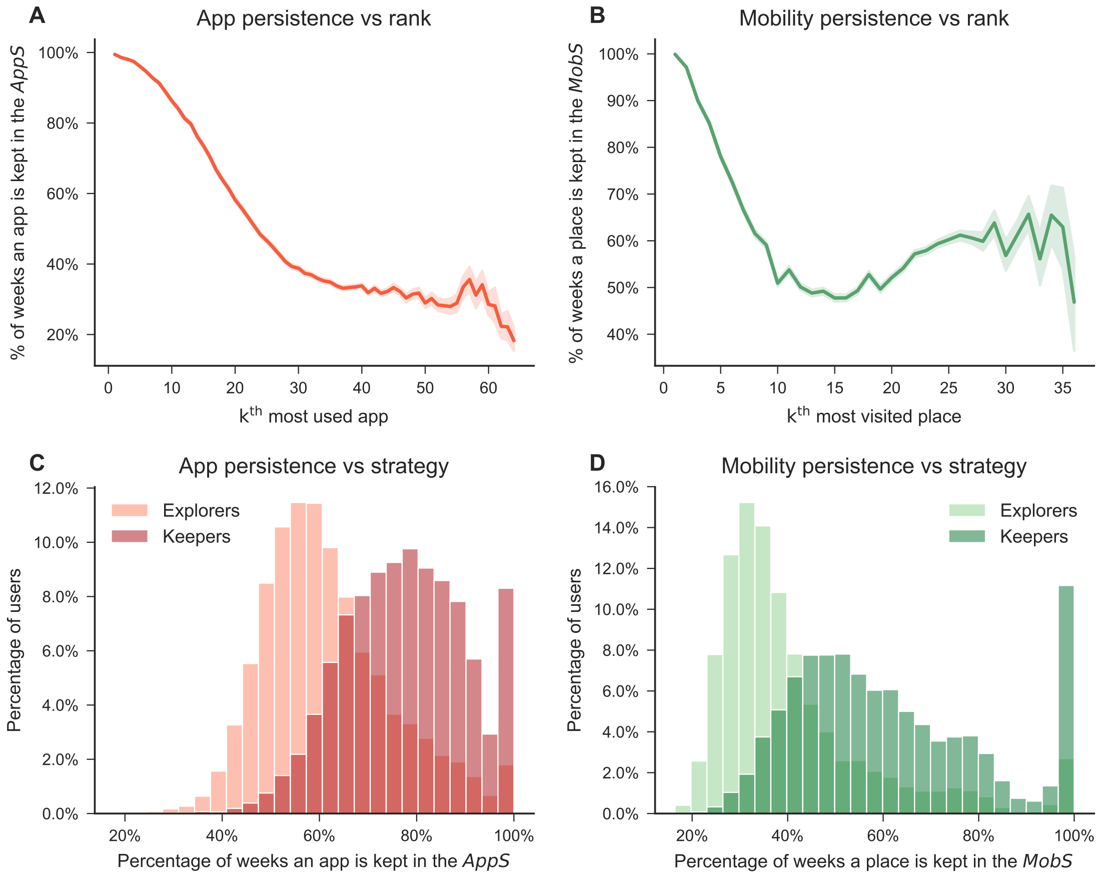

Figure S10: Percentage of time an app (left) or a location (right) is kept in the app (left) or activity (right) space once it enters, depending on the rank and strategy. (A-B) Distribution of the percentage of time an app (A) and a location (B) is kept in the app/activity space once it enters depending on its rank. (C-D) Distribution of the percentage of time an app (C) and a location (D) is kept in the app/activity space once it enters for Explorers and Keepers. Note that apps are kept for longer than locations.

4 Additional tables

Table S2: Most persistent mobile apps, i.e., mobile applications kept for the longest amount time, among those entered in the app space and used by at least 5% of the people.

Package name	Common name	% weeks	% of users
com.whatsapp	Whatsapp	97.68	91.94
com.facebook.orca	Messanger	96.57	84.45
com.facebook.katana	Facebook	96.24	90.80
com.touchtype.swiftkey	SwiftKey Keyboard	95.65	9.42
com.android.chrome	Chrome	93.87	87.97
com.google.android.apps.messaging	Messages	93.85	7.32
com.yahoo.mobile.client.android.mail	Yahoo Mail	92.50	11.54
com.bskyb.skynews.android	Sky News	90.66	6.48
com.rbs.mobile.android.natwest	NatWest Mobile Banking	89.97	14.19
com.grppl.android.shell.CMBlloydsTSB73	Lloyds Bank Mobile Banking	89.22	12.68
bbc.mobile.news.uk	BBC News	88.92	16.05
com.instagram.android	Instagram	88.72	65.14
com.microsoft.office.outlook	Outlook	88.34	26.32
com.snapchat.android	Snapchat	87.85	41.90
uk.co.santander.santanderUK	Santander Mobile Banking	87.63	9.82
com.bskyb.sportnews	Sky Sports	87.43	9.56
com.grppl.android.shell.halifax	Halifax	87.30	13.77
com.bskyb.fbscore	Sky Sports Football Score Centre	86.66	6.74
com.twitter.android	Twitter	86.66	32.81
com.google.android.youtube	Youtube	86.41	90.79

Table S3: Least persistent mobile apps, i.e., mobile applications kept for the least amount of time, among those entered in the app space and used by at least 5% of the people.

Package name	Common name	% weeks	% of users
com.bitstrips.imoji	Bitmoji	31.35	7.02
air.ITVMobilePlayer	ITV Hub	36.61	7.23
com.google.android.play.games	Google Play Games	38.97	12.47
com.sec.android.gallery3d	Samsung Gallery	41.64	31.95
com.channel4.ondemand	Channel 4 Television Corporation	43.14	5.41
com.google.android.apps.plus	Google+ for G Suite	45.13	6.12
uk.co.theofficialnationallotteryapp.android	The Official National Lottery	45.15	6.03
com.google.android.apps.walletnfcrel	Wallet	45.31	7.05
com.google.android.apps.docs.editors.docs	Google Docs	45.68	6.38
com.google.android.music	Google Play Music	45.99	20.98
com.amazon.dee.app	Amazon Alexa	47.57	6.76
bbc.iplayer.android	BBC iPlayer	47.79	11.02
com.paypal.android.p2pmobile	PayPal	48.81	14.65
com.samsung.android.spay	Samsung Pay	49.69	19.76
com.samsung.android.oneconnect	SmartThings	50.51	44.81
com.ubercab.eats	Uber Eats	52.14	5.21
com.sec.android.app.music	Samsung Music	53.15	15.55
com.myfitnesspal.android	Calorie Counter - MyFitnessPal	53.26	7.24
uk.co.dominos.android	Domino's Pizza	53.38	7.47
com.booking	Booking	53.81	11.09

Table S4: Most frequently dropped apps.

Package name	Common name	Number drops
com.sec.android.inputmethod	Samsung Keyboard	17928
com.samsung.knox.securefolder	Secure Folder	17310
com.samsung.android.oneconnect	SmartThings	14456
com.google.android.apps.docs	Docs	11895
com.google.android.gm	Gmail	9790
com.google.android.apps.maps	Maps	6526
com.google.android.youtube	Youtube	6325
com.microsoft.office.powerpoint	Powerpoint	4513
com.sec.android.gallery3d	Samsung Gallery	4376
com.google.android.apps.photos	Photos	4259

Table S5: Most frequently dropped categories of apps.

Rank	Category name
1	Productivity
2	Lifestyle
3	Communication
4	Shopping
5	Entertainment
6	Business
7	Travel & Local
8	Social
9	Finance
10	Music & Audio

Table S6: Classification performances of baseline model, Random Forest and multinomial Logistic regression model. Performances are expressed as micro-averaged and macro-averaged multi-class F1-scores.

Model	F1 macro	F1 micro
**Mobility to App**		
Baseline (Dummy)	0.33	0.44
Logistic	0.30	0.31
Random Forest	0.39	0.54
**App to Mobility**		
Baseline (Dummy)	0.33	0.44
Logistic	0.23	0.23
Random Forest	0.39	0.53

References

[1] L. Alessandretti, P. Sapiezynski, V. Sekara, S. Lehmann, and A. Baronchelli. Evidence for a conserved quantity in human mobility. *Nature Human Behaviour*, 2(7):485–491, 2018.

[2] M. Ester, H.-P. Kriegel, J. Sander, and X. Xu. A density-based algorithm for discovering clusters a density-based algorithm for discovering clusters in large spatial databases with noise. In *Proceedings of the Second International Conference on Knowledge Discovery and Data Mining*, KDD'96, pages 226–231. AAAI Press, 1996.

[3] R. Hariharan and K. Toyama. Project lachesis: Parsing and modeling location histories. In M. J. Egenhofer, C. Freksa, and H. J. Miller, editors, *Geographic Information Science*, pages 106–124, Berlin, Heidelberg, 2004. Springer Berlin Heidelberg.

[4] K. Katevas, I. Arapakis, and M. Pielot. Typical phone use habits: Intense use does not predict negative well-being. In *Proceedings of the 20th International Conference on Human-Computer Interaction with Mobile Devices and Services*, MobileHCI '18, pages 11:1–11:13, New York, NY, USA, 2018. ACM.

[5] M. G. Kendall. A new measure of rank correlation. *Biometrika*, 30(1/2):81–93, 1938.

[6] E. L. Kenney and S. L. Gortmaker. United states adolescents' television, computer, videogame, smartphone, and tablet use: associations with sugary drinks, sleep, physical activity, and obesity. *The Journal of pediatrics*, 182:144–149, 2017.

[7] L. Pappalardo, F. Simini, S. Rinzivillo, D. Pedreschi, F. Giannotti, and A.-L. Barabási. Returners and explorers dichotomy in human mobility. *Nature communications*, 6:8166, 2015.

[8] L. Yang, M. Yuan, W. Wang, Q. Zhang, and J. Zeng. Apps on the move: A fine-grained analysis of usage behavior of mobile apps. In *IEEE INFOCOM 2016 - The 35th Annual IEEE International Conference on Computer Communications*, pages 1–9, April 2016.
